# Genetically modified artificial antigen-presenting cells (aAPC) for expansion of melanoma tumor infiltrating lymphocytes with optimal properties for adoptive cell therapy

**DOI:** 10.1186/2051-1426-1-S1-P8

**Published:** 2013-11-07

**Authors:** Marie-Andrée Forget, Shruti Malu, Hiu Liu, Christopher Toth, Sourindra Maiti, Charuta Kale, Chantale Bernatchez, Helen Huls, Ena Wang, Patrick Hwu, Laurence J Cooper, Laszlo G Radvanyi

**Affiliations:** 1 Melanoma Medical Oncology, MD Anderson Cancer Center, Houston, TX, USA; 2 Transfusion Medicine, National Institutes of Health, Bethesda, MD, USA; 3 Pediatrics Research, MD Anderson Cancer Center, Houston, TX, USA

## 

Adoptive cell therapy (ACT) with expanded autologous tumor infiltrating lymphocytes (TIL) has emerged to be a powerful salvage therapy for metastatic melanoma with response rates up to 50%. However, the generation of the TIL ACT product is technically challenging with current methods requiring a large excess of allogeneic normal donor (pool) or patient-derived PBMC (200:1 ratio to the TIL) used as “feeders” to support a rapid expansion protocol (REP) to generate the final TIL infusion product. Because PBMC feeder products consist of a heterogenous cell population, it introduces undesired variability in TIL expansion rates and phenotype, especially in the yield of CD8+ T cells that are the most active component of the TIL product. Here, we have developed an alternative to PBMC feeders using a K562-based artificial antigen-presenting cell (aAPC) system expressing CD64, CD86, and 4-1BBL that is much more practical and cost-effective. Using PBMC feeders as controls, we found an optimal aAPC:TIL ratio (50:1) supporting maximal TIL expansion used for subsequent experiments. Analysis of the resulting TIL products from multiple experiments revealed that the aAPC induced higher rates of CD8+ T cell expansion with a comparable effector-memory phenotype as TIL expanded with the traditional PBMC feeder approach. The exceptions were CD56 that was more highly expressed on the CD8+ cells and CD28 which had a lower expression. Notably, TIL expanded with aAPC were enriched in CD8+ BTLA+ T cells, a subset highly correlated with clinical response to ACT. TCR Vαβ clonotype analysis also found that TIL expansion with the aAPC did not alter the diversity of the T cell repertoire. Further analysis using gene chip profiling revealed significant differences in gene expression in TIL products expanded using the aAPC versus with PBMC feeders. These included an up-regulation of certain genes in the Wnt pathway, cyclic nucleotide metabolism, and multiple genes in different stem cell pathways. This more stem-like profile may have beneficial properties following adoptive transfer. Finally, we found that CD8+ TIL expanded with aAPC had a similar anti-tumor effector cell activity in CTL and IFN-γ release assays. Overall, our data demonstrates that this aAPC system is a suitable alternative to generate clinical-grade melanoma TIL infusion products for ACT that produces comparable and perhaps even more active T cells than current methods. Our group is currently phasing in the use of these aAPC for our GMP TIL production.

